# Cyclodextrins Exert a Ligand-like Current Inhibitory Effect on the K_V_1.3 Ion Channel Independent of Membrane Cholesterol Extraction

**DOI:** 10.3389/fmolb.2021.735357

**Published:** 2021-11-04

**Authors:** Tamas Kovacs, Tamas Sohajda, Lajos Szente, Peter Nagy, Gyorgy Panyi, Zoltan Varga, Florina Zakany

**Affiliations:** ^1^ Division of Biophysics, Department of Biophysics and Cell Biology, Faculty of Medicine, University of Debrecen, Debrecen, Hungary; ^2^ CycloLab Cyclodextrin R and D Laboratory Ltd., Budapest, Hungary

**Keywords:** cyclodextrin, cholesterol, membrane fluidity, membrane hydration, membrane lipid order, ligand-like interaction, K_V_1.3

## Abstract

Cyclodextrins (CDs) are cyclic oligosaccharides capable of forming water-soluble complexes with a variety of otherwise poorly soluble molecules including cholesterol and different drugs. Consistently, CDs are widely used in research and clinical practice to deplete cholesterol from cellular membranes or to increase solubility and bioavailability of different pharmaceuticals at local concentrations in the millimolar range. Effects of CDs exerted on cellular functions are generally thought to originate from reductions in cholesterol levels. Potential direct, ligand-like CD effects are largely neglected in spite of several recent studies reporting direct interaction between CDs and proteins including AMP-activated protein kinase, β-amyloid peptides, and α-synuclein. In this study, by using patch-clamp technique, time-resolved quantitation of cholesterol levels and biophysical parameters and applying cholesterol-extracting and non-cholesterol-extracting CDs at 1 and 5 mM concentrations, we provide evidence for a previously unexplored ligand-like, cholesterol-independent current inhibitory effect of CDs on K_V_1.3, a prototypical voltage-gated potassium channel with pathophysiological relevance in various autoimmune and neurodegenerative disorders. Our findings propose that potential direct CD effects on K_V_ channels should be taken into consideration when interpreting functional consequences of CD treatments in both research and clinical practice. Furthermore, current-blocking effects of CDs on K_V_ channels at therapeutically relevant concentrations might contribute to additional beneficial or adverse effects during their therapeutic applications.

## Introduction

Cyclodextrins (CDs) are cyclic oligosaccharides typically consisting of six (αCD), seven (βCD) or eight (γCD) alpha-D-glucopyranoside units (Davis and Brewster, 2004; Uekama, 2004). Their fundamental spatial structure exhibits a truncated cone-shaped conformation in which the degree of polymerization defines the size and the side-chain substitutions influence the hydrophobicity of their cavity. The most commonly applied CDs have a hydrophobic internal cavity and a hydrophilic outer surface, which creates a suitable milieu for making reversible water-soluble non-covalent complexes with a wide range of poorly soluble molecules including cholesterol and various drugs (Davis and Brewster, 2004; Uekama, 2004; Loftsson et al., 2005; Szente and Fenyvesi, 2017; Szente et al., 2018). Up to 100 medications currently available worldwide contain CDs as a main active agent to deplete cholesterol or as adjuvants in formulations to increase solubility and bioavailability of different pharmaceuticals.

In research, randomly-methylated-β-cyclodextrin (MβCD) is most widely used due to its highest efficiency among CD derivatives to extract cholesterol from the cell membrane (Szente and Fenyvesi, 2017; Szente et al., 2018). It is typically applied at 3–5 mM for 1 h for membrane cholesterol depletion, while MβCD previously complexed with cholesterol potently elevates membrane cholesterol levels (Zidovetzki and Levitan, 2007; Bukiya et al., 2021). In clinical practice and *in vivo* experiments, hydroxypropyl-β-cyclodextrin (HPβCD) is the most commonly employed CD to deplete cholesterol due to its better safety profile compared to MβCD (Irie and Uekama, 1997; Szente et al., 2018). When administered intravenously or intracerebroventricularly for the treatment of Niemann-Pick type C (NPC) disease, a rare lysosomal storage disorder characterized by cellular cholesterol accumulation, local HPβCD concentrations can reach concentrations in the millimolar range (Megias-Vericat et al., 2017; Carradori et al., 2020).

**FIGURE 1:**
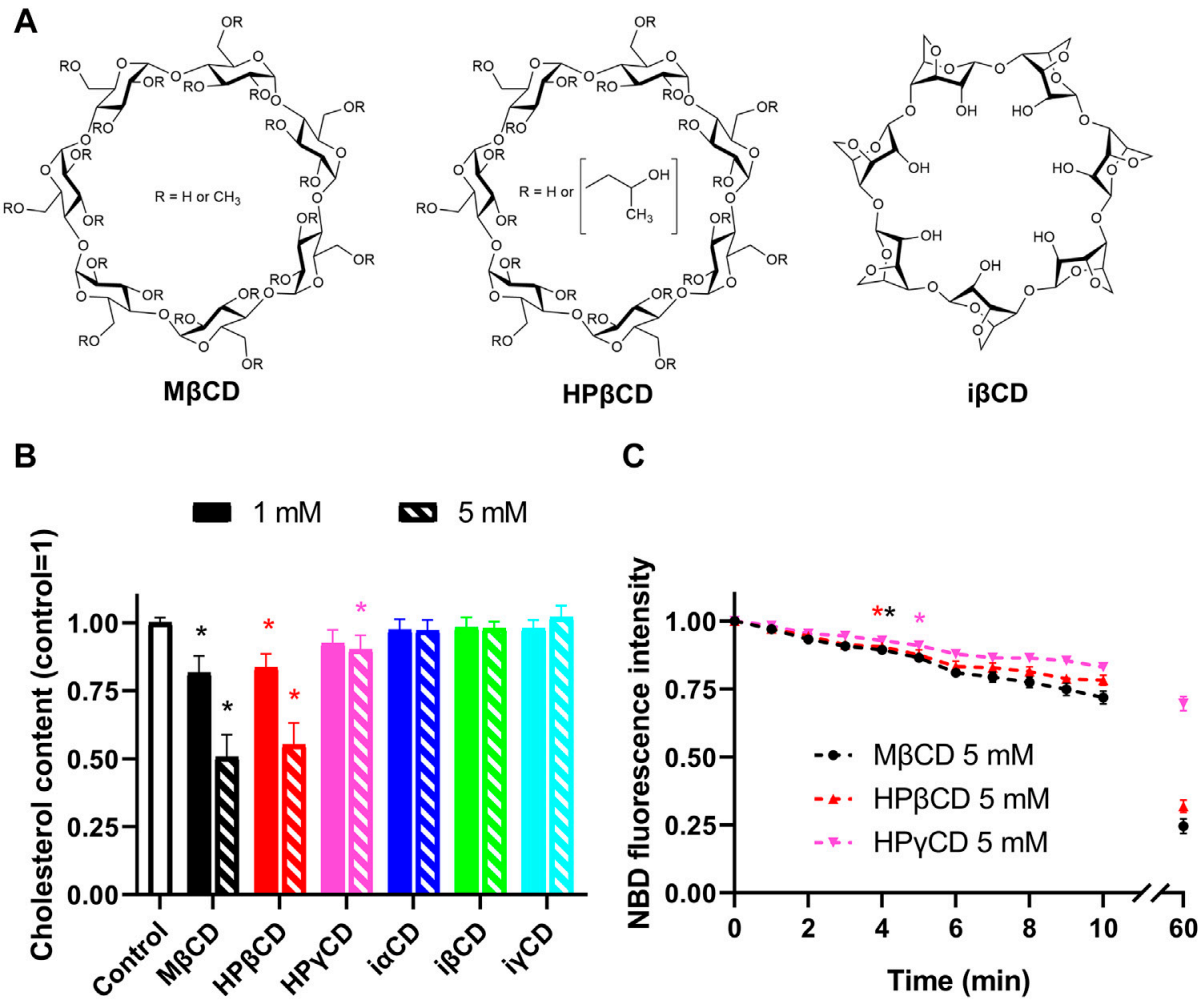
Chemical structures and membrane cholesterol-extracting effects of CDs. **(A)** CDs typically consist of six, seven, or eight alpha-D-glucopyranoside units, which are referred to as α-, β-, and γCDs. When comparing ring-like structures of conventional and “inverted” Per(3,6-anhydro)-CDs, the former are characterized by a hydrophobic internal cavity and a hydrophilic outer surface, and different side-chain substitutions fine-tune their chemical properties. MβCD, the most commonly applied derivative for cholesterol depletion *in vitro*, is randomly methylated as indicated by R groups in the figure, while HPβCD, the CD most widely used for cholesterol extraction *in vivo*, contains random substitutions with hydroxypropyl groups in these positions. On the other hand, iCDs such as iβCD are single isomers not modified on their glucose hydroxyl groups but their glucose core is itself modified, i.e., the ^4^C_1_ chair conformation of the natural glucopyranose units are transformed into ^1^C_4_ form. This modification of the conformation of glucose units causes a dramatic change in lipophilicity profile, as these compounds are characterized by a hydrophilic interior and a hydrophobic outer surface leading to preferential entrapment of hydrophilic guests. **(B)** CHO cells were treated with 1 or 5 mM CDs for 1 h followed by determination of cholesterol levels using fluorometric cholesterol quantitation kit. While conventional CDs induced dose-dependent decreases in membrane cholesterol contents according to the efficacy order MβCD > HPβCD > HPγCD, iCDs did not reduce cholesterol levels. **(C)** Kinetics of cholesterol extraction in response to CDs was examined after pre-loading CHO cells with 200 µM NBD-conjugated cholesterol complexed with MβCD for 1 h. Then, time-dependent changes in NBD fluorescence intensities induced by CDs were measured using time-resolved flow cytometry. Treatments started in the sample holder of the flow cytometer immediately before initiation of measurements. Moving averages of time-correlated fluorescence intensity values of approximately 300,000 cells per sample were calculated with a window size of 20 s and subsequently normalized to average intensities determined in the first time window. Time-dependent reductions in fluorescence intensities were observed in response to all three examined conventional CDs with the efficacy order MβCD > HPβCD > HPγCD. First significant changes appeared 240, 220, and 300 s after the initiation of treatments, respectively. Data are represented as mean ± SEM obtained from *n* = 6 independent experiments. Asterisks (*) indicate significant differences compared to control samples (*p* < 0.05, ANOVA followed by Tukey’s HSD test). For clarity, in panel B every third point is shown and the first time point is marked by an asterisk where significant difference is observed in response to the given treatment.

Interestingly, hydroxypropyl-γ-cyclodextrin (HPγCD), another biologically relevant CD derivative is also capable of decreasing cellular cholesterol levels despite its much lower affinity to form complexes with cholesterol *in vitro* (Szente et al., 2018; Yamada et al., 2021). This finding might draw attention to alternative CD-mediated cellular actions rather than solely focusing on cholesterol complexation. Per(3,6-anhydro)-CDs represent a scarcely known subfamily of CDs, which, as opposed to the original compounds, are characterized by an “inverted” structure with a hydrophilic interior and a hydrophobic outer surface (Yamamura et al., 1993; Ashton et al., 1996) (Figure 1A). Anhydro CDs are modified not on hydroxyls but on the glucose core. The ^4^C_1_ chair conformation of the glucopyranose units is transformed into ^1^C_4_ form, resulting in this inverted unique structure. Therefore, these inverted cyclodextrin (iCD) derivatives are unlikely to form complexes with cholesterol, however, their cholesterol extracting efficacies have not been characterized yet.

Recently direct, ligand-like interactions have been demonstrated between CDs and proteins including α-synuclein (Gautam et al., 2014), β-amyloid peptides (Wahlström et al., 2012; Ren et al., 2016), prion proteins (Prior et al., 2007) and AMP-activated protein kinase (Dai et al., 2017), which suggests that CDs might be able to affect the functions of proteins independently of their membrane cholesterol depleting effects as well. Ionic currents of voltage-gated potassium channels (K_V_) are largely influenced by the direct binding of peptide toxins and small molecule inhibitors (Tajti et al., 2020). The actions of these molecules are extensively studied on K_V_1.3, a channel with structural properties and gating mechanisms prototypical for most members of the K_V_ family (Liu et al., 2021), and essential functional roles in lymphocytes and microglial cells thus representing an attractive therapeutic target in many autoimmune and neurodegenerative disorders (Feske et al., 2015; Sarkar et al., 2020; Ramesha et al., 2021). The operation of K_V_ channels is also sensitive to levels of membrane lipids including cholesterol (Bock et al., 2003; Lipinsky et al., 2020; Zakany et al., 2020). It has been shown that 1-h incubation with 3–5 mM MβCD and subsequently decreased cholesterol levels of the cell membrane result in an increase in K_V_1.3 current amplitude, while membrane cholesterol loading with MβCD-cholesterol complexes has opposing effects (Hajdú et al., 2003; PottosinValencia-Cruz et al., 2007; Balajthy et al., 2016; Zakany et al., 2019). While most of these studies shedding light on cholesterol effects on K_V_1.3 were carried out with CDs, direct interactions between CDs and K_V_ channels have not been investigated previously.

Here, we report on a previously unexplored, direct, ligand-like inhibitory effect of CDs on K_V_1.3 current. Applying inverted, non-cholesterol-extracting per (3,6-anhydro)-CDs as tools for the exclusive examination of ligand-like CD effects during electrophysiological experiments, and performing time-resolved measurements to quantify cholesterol extraction and alterations in membrane fluidity, we demonstrate that this current inhibiting effect is independent of membrane cholesterol depletion and not related to alterations induced in membrane biophysical parameters. *In silico* molecular docking analysis further supported the presence of direct interactions between CDs and the ion channel. Our findings emphasize that potential direct CD effects on K_V_ channels should be taken into consideration when interpreting functional consequences of CD treatments in both research and clinical practice. Furthermore, current blocking effects of CDs at therapeutically relevant concentrations in the millimolar range might contribute to additional beneficial or adverse effects during their medical applications.

## Materials and Methods

### Cell Culture, Transfection and Cyclodextrins

Chinese hamster ovary (CHO) cells were obtained from the American Type Culture Collection (Manassas, VA) and transfected with wild-type K_V_1.3 and enhanced green fluorescent protein (EGFP) encoding plasmids (OriGene Technologies, MD, United States) using Lipofectamine 2000 (Thermo Fisher Scientific, Waltham, MA) as described in Supplementary Material.

Methyl-β-cyclodextrin (MβCD), hydroxypropyl-β-cyclodextrin (HPβCD), hydroxypropyl-γ-cyclodextrin (HPγCD), and “inverted” cyclodextrins (iCDs) including hexakis (3,6-anhydro)-α-cyclodextrin (iαCD), heptakis (3,6-anhydro)-β-cyclodextrin (iβCD) and octakis (3,6-anhydro)-γ-cyclodextrin (iγCD) were obtained from CycloLab Cyclodextrin R&D Laboratory (Budapest, Hungary). For further characterization of iCDs, see Supplementary Methods. In general, cells were treated with CDs dissolved in standard extracellular solution at concentrations of 1 or 5 mM at room temperature. Incubation times and other details of CD applications for the given experiments are described in the following sections.

### Quantification of Cellular Cholesterol Content

Cholesterol contents of control samples and those treated with different CDs for 1 h were determined using a fluorometric cholesterol quantitation kit (Sigma-Aldrich) according to instructions of the manufacturer with a Synergy HT Microplate Reader (BioTek Instruments, Winooski, VT, United States). For kinetic examination of membrane cholesterol extraction, cells were pre-loaded using MβCD complexed with NBD-conjugated cholesterol (25-[N-[(7-nitro-2-1,3-benzoxadiazol-4-yl)methyl]amino]-27-norcholesterol, Avanti Polar Lipids, Alabaster, AL and CycloLab Cyclodextrin R&D Laboratory) for 1 h at a concentration of 195 µM sterol, followed by incubation with 1 or 5 mM of CDs in the sample holder of a NovoCyte 3000RYB flow cytometer (ACEA Biosciences, San Diego, CA) at room temperature. NBD fluorescence intensity was determined using excitation at 488 nm and an 530/30 nm emission filter. Measurements started immediately after addition of CDs and continued for 10 min. Time-correlated fluorescence intensity values were quantified with FCS Express (De Novo Software, Pasadena, CA) and a custom-written Matlab program as described previously (Batta et al., 2021).

### Patch-Clamp Measurements

Patch-clamp measurements were carried out in whole-cell or outside-out configuration using a KF-based pipette solution with a final K^+^ concentration of 160–165 mM and a standard extracellular solution containing 150 mM NaCl (see also Supplementary Material). CDs were dissolved in standard extracellular solution that were directly applied on the cells with a gravitation propelled perfusion system. The proper working of the perfusion system was validated by applying a high potassium (150 mM KCl) containing extracellular solution before the measurement of every single cell to exclude false negative cases originating from the inappropriate flow of CD-containing solutions.

For characterizing direct inhibitory effects of CDs, 15 ms depolarizing pulses to +50 mV were applied every 15 s from a holding potential of –100 mV. The current inhibitory effects of CDs at given concentrations were determined as remaining current fractions (RCF) using the following equation

$$\text{RCF}=\frac{I}{I_{0}}$$ (1)

where *I*_*0*_ is the leak-corrected peak current in standard extracellular solution before CD applications and *I* is the leak-corrected peak current of the same patch at equilibrium block, at a given CD concentration. To demonstrate the washing-in kinetics of CDs, leak-corrected peak currents at every time point were normalized to the maximal peak. To characterize the extent of washing-out of CDs, recovered current fractions (RF) were determined as a leak-corrected peak current in standard extracellular solution after washing-out the given CDs (I_CD_) over the initial leak-corrected peaks (I_0_) before CD applications according to

$$\text{RF}=\frac{I_{\text{CD}}}{I_{0}}$$ (2)

### Examination of Membrane Biophysical Parameters

To measure changes in membrane fluidity, hydration and lipid order in response to CDs, fluorescence anisotropy of 4′-(trimethylammonio)-diphenylhexatriene (TMA-DPH) and generalized polarization (GP) of two indicators, 6-dodecanoyl-N,N-dimethyl-2-naphthylamine (Laurdan) and 4-[2-(6-Dibutylamino-5-fluoro-naphthalen-2-yl)-vinyl]-1-(3-triethylammonio-propyl)-pyridinium dibromide (PY3174), were quantified as described previously and in Supplementary Material (Kwiatek et al., 2013; Kovács et al., 2017; Zakany et al., 2021).

### In silico Molecular Docking Analysis

*In silico* docking was performed with AutoDock Vina (Trott and Olson, 2009). The conformation of MβCD was extracted from the crystal structure of the extracellular domain of human Gastric inhibitory polypeptide receptor (PDB 2QKH) that was cocrystallized with MβCD (Parthier et al., 2007). The target of docking was the cryo-electron microscopic structure of K_V_1.3 (PDB 7EJ1) (Liu et al., 2021) and the search space was defined to include the extracellular orifice of the pore domain (Supplementary Figure S1). AutoDock Vina was run with an exhaustiveness parameter of 40, and ten binding modes were recorded. Amino acids taking part in ligand-target interactions were identified and displayed using LigPlot+ (Laskowski and Swindells, 2011) and PyMol.

### Statistical Analysis

Measured data are represented as mean ± SEM obtained from *n* independent samples indicated in figure legends. Differences were considered significant (*) when *p* < 0.05 calculated based on ANOVA followed by Tukey’s HSD test.

## Results

### Cholesterol-Extracting Efficiencies and Kinetics of Cyclodextrins

In order to test membrane cholesterol depletion induced by CDs with different cavity charge profiles (Figure 1A), we examined cellular cholesterol levels using a commercially available fluorometric cholesterol quantitation kit after treatment of CHO cells with 1 and 5 mM of CDs for 1 h. In CDs with conventional cavity polarity we obtained data consistent with literature (Zidovetzki and Levitan, 2007; Szente et al., 2018). MβCD and HPβCD, two derivatives most widely used and most effective in cholesterol extraction, induced significant and comparable dose-dependent decreases in cholesterol levels reaching ∼50% extraction at a concentration of 5 mM (Figure 1B). HPγCD with much lower affinity for forming complexes with cholesterol also resulted in reduced cholesterol contents, however, these reductions were much smaller thus reaching the level of significance only at 5 mM. Concordant with our expectations, iCDs with a hydrophilic cavity did not cause any significant alterations in membrane cholesterol levels.

To characterize the time dependence of cholesterol extraction induced by MβCD, HPβCD, and HPγCD, we preloaded cells with NBD-cholesterol, a fluorophore-conjugated sterol derivative, and examined changes in fluorescence intensities of cells using time-resolved flow cytometry. Treatments with 5 mM of CDs started in the sample holder of the flow cytometer immediately before the initiation of measurements. With this technique we were able to follow reductions in levels of exogenous cholesterol molecules incorporated into cellular membranes with a time resolution of 20 s in the first 10 min of treatments. We observed time-dependent decreases in fluorescence in response to all three examined CDs (Figure 1C). Again, consistent with literature, cholesterol-extracting abilities of MβCD and HPβCD were superior to that of HPγCD (Zidovetzki and Levitan, 2007; Szente et al., 2018). Since only ∼12% of the cholesterol extracted by a 1-h CD treatment was depleted from cells in the first 3 min, these experiments suggested that cholesterol depletion in response to conventional CDs is dominant mainly after longer incubation periods. Considering a recent report suggesting similar efficiency of MβCD to extract unlabeled and NBD-cholesterol in living cells (Ostašov et al., 2013), this piece of information was utilized for the design of patch-clamp measurements to exclusively separate in time the direct, ligand-like and indirect effects of CDs showing significant cholesterol-depleting abilities.

### Effects of Cyclodextrins on K_V_1.3 Current

**FIGURE 2:**
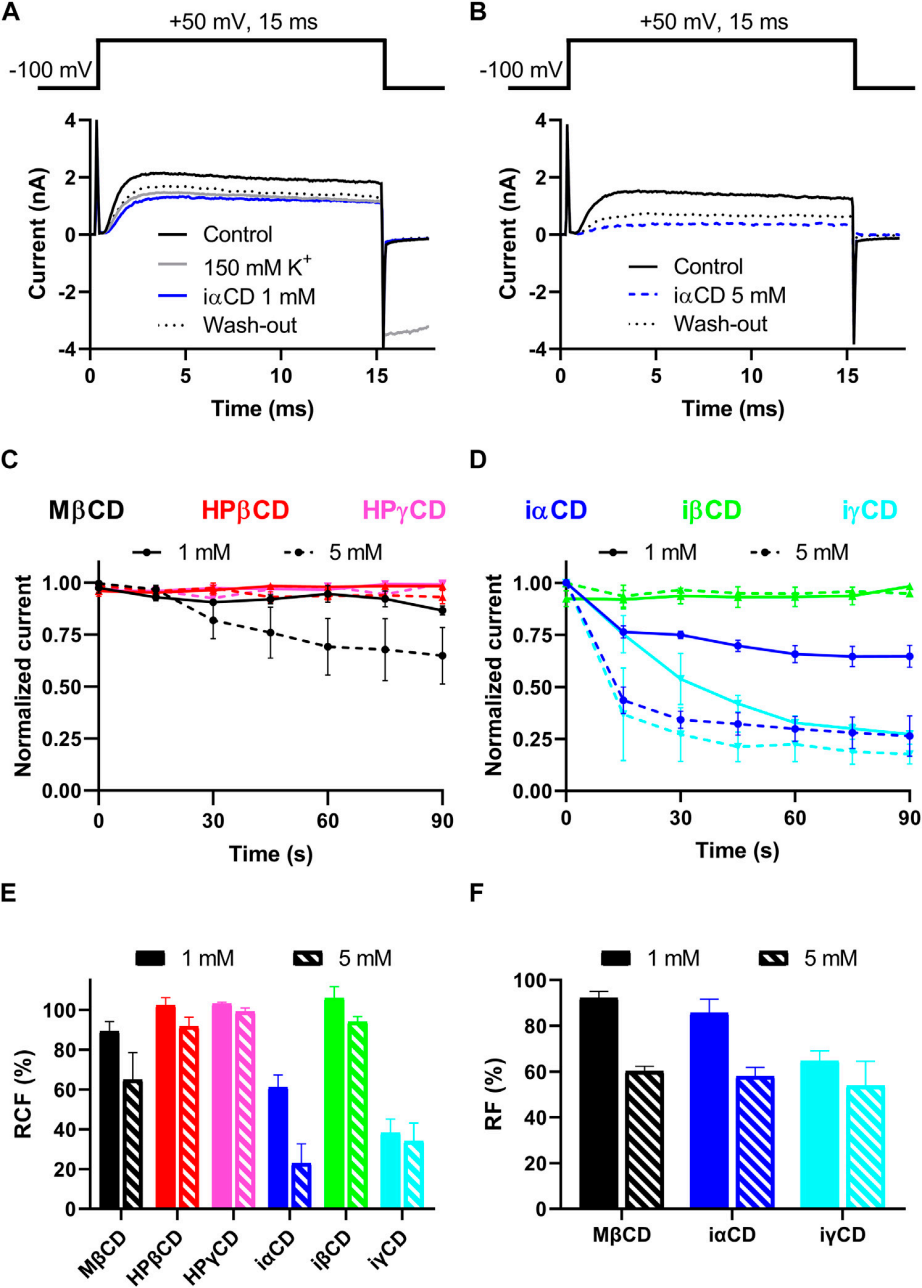
Effects of CDs on K_V_1.3 current. **(A,B)** Patch-clamp measurements were performed in outside-out or whole-cell configurations in CHO cells transiently expressing K_V_1.3. Two representative measurements with the application of 1mM (panel A) and 5mM (panel B) iαCD are shown. K^+^ currents were elicited by applying 15ms-long depolarizing pulses from −100mV to +50mV every 15s (panels A and B, top). First, cells were perfused with standard extracellular solution to obtain control currents (*I*_*0*_, panels A and B, black solid lines). To validate proper working of the perfusion system an extracellular solution with 150mM K^+^ was applied for each cell prior to CD exposures (panel A, grey line). Cells were perfused with CD-containing extracellular solutions for a maximal application time of 3min to avoid cholesterol-extracting effects of CDs (panels A and B blue lines). Then the reversibility of current-blocking effects induced by the compounds was determined by the reapplication of standard extracellular solution (panel A and B black dotted lines). **(C,D)** To demonstrate the wash-in kinetics of CDs, leak-corrected peak currents at every time point were normalized to the maximal peak. Among cholesterol-depleting CDs, only MβCD in 5mM exhibited a current block (Panel C, black dashed line), while among the non-cholesterol depleting inverted CDs, iαCD (panel D, blue lines) and iγCD (panel D, cyan lines) showed similar effects at both 1mM (solid lines) and 5mM (dashed lines) concentrations. The current blocking effects were saturated within 90s after the initiation of CD exposure in all cases. **(E)** To quantify current-inhibiting effects, remaining current fraction (RCF) values were calculated according to equation (Davis and Brewster, 2004). The efficacy order of the induced current block was MβCD < iαCD < iγCD in both concentrations. **(F)** To characterize the extent of wash-out, recovered current fractions (RF) were determined according to equation (Uekama, 2004). This analysis showed that the current block induced by CDs was only partially reversible. Data represented as mean ± SEM based on *n* = 4–6 cells.

The potential direct, ligand-like effect of CDs on K_V_1.3 was tested by patch-clamp. Currents were elicited by 15-ms depolarizing steps to +50 mV from a holding potential of –100 mV (Figures 2A,B, top panels) to fully activate K_V_1.3 current (Figures 2A,B, bottom panel, black solid lines). Only those experiments were evaluated, where the proper working of the perfusion apparatus was verified by changes in current parameters in response to high potassium (150 mM) solution (Figure 2A, grey solid line), which could be subsequently reverted in one step by re-applying standard extracellular solution (not shown for clarity). The exclusive investigation of direct, ligand-like interactions between CDs and K_V_1.3 was ensured in two ways. 1) If patch-clamp measurements are performed in 3 minutes after applying the CD-containing extracellular solutions, no significant cholesterol extraction in response to the cholesterol-depleting CDs takes place (Figure 1B). 2) Application of iαCD, iβCD and iγCD is a suitable tool for the exclusive examination of direct ligand-like effects of CDs since these derivatives are not able to deplete membrane cholesterol even after 1 h (Figure 1A). As indicated by representative current traces (Figures 2A,B, blue traces) and decreases in RCF values (Figure 2E), a current block can be detected in the presence of both 1 and 5 mM iαCD. Similarly, MβCD and iγCD also resulted in dose-dependent current blocking effects, as shown by decreases in RCF values, while neither the cholesterol-depleting HPβCD and HPγCD, nor the non-cholesterol-depleting iβCD induced any remarkable changes in RCF (Figure 2E). According to the wash-in kinetics for MβCD (Figure 2C, black lines), iαCD and iγCD (Figure 2D blue and cyan lines), current-blocking effects were completed within 90 s after the initiation of CD exposure in the absence of significant cholesterol depletion according to Figure 1B. In all three cases, current blocking effects were only partially reversible (Figures 2A,B dotted lines), as indicated by RF values (Figure 2F).

### Alterations in Membrane Biophysical Parameters Induced by Cyclodextrins

**FIGURE 3:**
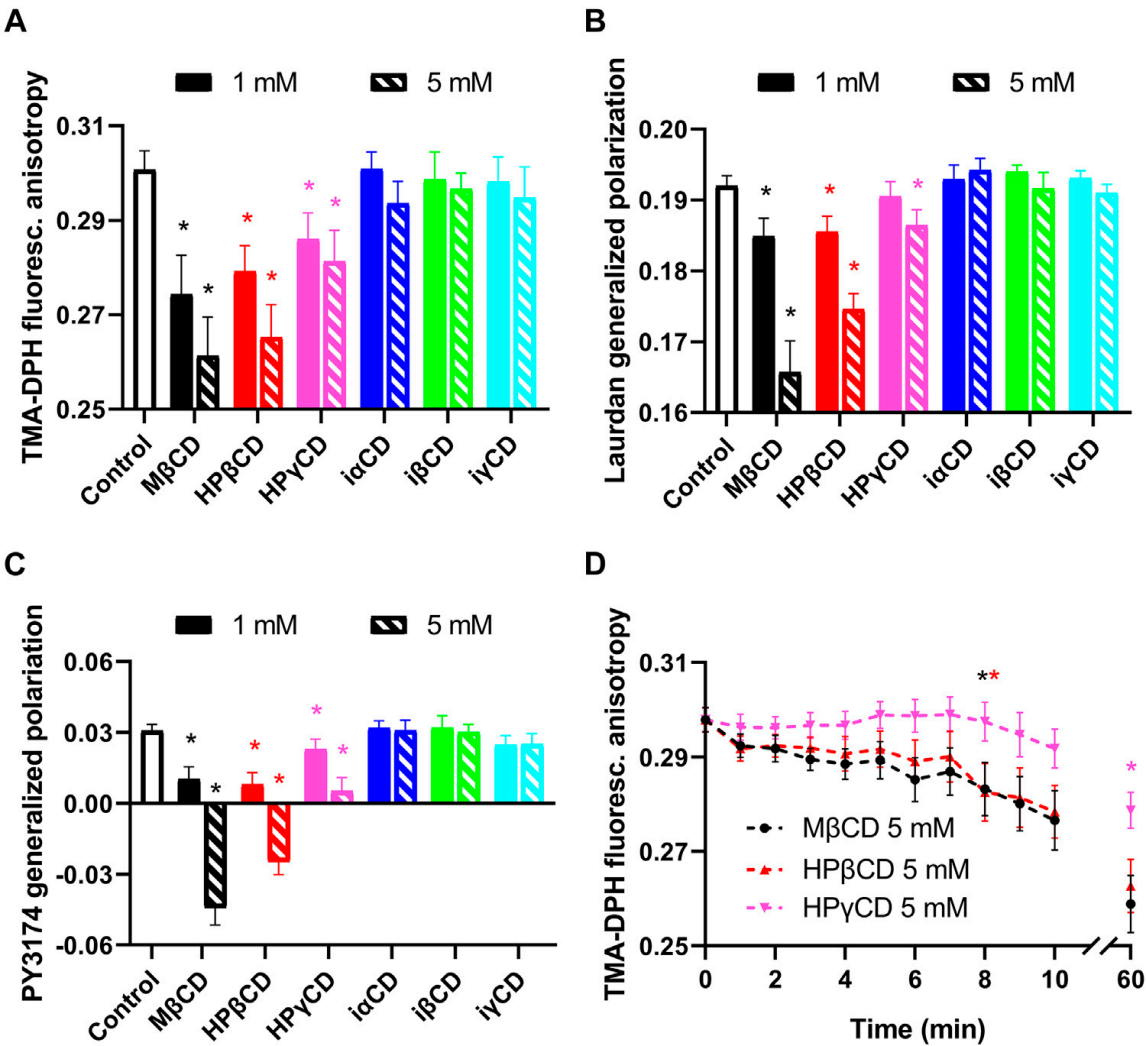
Effects of CDs on membrane biophysical parameters. **(A)** CHO cells were treated with 1 or 5 mM CDs for 1 h, labeled with TMA-DPH and the fluorescence anisotropy of the fluorophore was determined using spectrofluorometry. **(B)** Cells treated as above were alternatively labeled with Laurdan, followed by quantification of generalized polarization (GP) of the dye with spectrofluorometry. **(C)** Control and treated cells were stained with PY3174 and the GP of the dye localized in the cell membrane was subsequently examined using confocal microscopy and quantitative image analysis. Cholesterol-extracting CDs affected biophysical parameters of the cell membrane in the efficacy order MβCD > HPβCD > HPγCD, as indicated by decreases in TMA-DPH anisotropy implying increased membrane fluidity (A), reductions in Laurdan GP and PY3174 GP referring to increased membrane hydration or lower membrane lipid order (B, C). **(D)** Kinetics of membrane fluidizing effects of CDs was examined after pre-staining CHO cells with TMA-DPH, which was followed by treating them with 5 mM of CDs in the cuvette of the spectrofluorometer. Treatments started immediately before initiation of measurements that included repeated quantification of fluorescence anisotropy of the dye every minute in the first 10 min of incubation and, as an end-point of the experiments, after 60 min. Time-dependent reductions in TMA-DPH anisotropy were observed in response to all three examined conventional CDs with the efficacy order MβCD > HPβCD > HPγCD. The first significant changes appeared in 8 min in response to MβCD and HPβCD, while in the case of HPγCD reductions were significant only after 60 min. In the figure, TMA-DPH anisotropy and Laurdan GP values are represented as mean ± SEM obtained from *n* = 9 independent samples containing approximately 100,000 cells. In panel C, mean PY3174 GP values ±SEM of 20 individual images obtained in five independent experiments are plotted for the different treatments. Each image contained data of 5–10 cells of normal morphology with a total number of 100–200 cells per treatment. Asterisks (*) indicate significant differences compared to control samples (*p* < 0.05, ANOVA followed by Tukey’s HSD test). For clarity, in panel D the first time point is marked by an asterisk where significant difference is observed in response to the given treatment.

Although experiments described in the previous section suggest direct interaction between CDs and K_V_1.3 ion channels, we examined whether CDs can possibly exert their effects through changes in biophysical properties of the cell membrane. Therefore, we examined membrane fluidity, hydration and lipid order using environment-sensitive fluorophores. First, to test membrane fluidity, we treated cells with 1 or 5 mM CDs for 1 h and determined TMA-DPH fluorescence anisotropy negatively correlating with the fluidity of the cell membrane (Batta et al., 2021; Zakany et al., 2021). As can be expected from the intimate relationship between membrane cholesterol content and fluidity, TMA-DPH fluorescence anisotropy of control cells was significantly decreased by cholesterol-extracting CDs (Figure 3A). On the other hand, iCDs not capable of depleting membrane cholesterol induced no significant changes in membrane fluidity. Similar effects were observed when examining membrane hydration through quantification of Laurdan GP inversely correlating with membrane hydration (Batta et al., 2021; Zakany et al., 2021). Laurdan GP of control cells was significantly reduced by CDs causing cholesterol depletion, while it was not affected by iCDs (Figure 3B). In order to remove the potential contribution of internalized Laurdan to the measured signal and to ascertain that the calculated GP values reflect the hydration of the plasma membrane where the potassium channels are expressed, membrane lipid order was also investigated with PY3174. Analysis of PY3174 GP, a parameter that positively correlates with the degree of lipid order (Kwiatek et al., 2013; Zakany et al., 2021), was restricted to the plasma membrane using segmentation of confocal microscopic images. PY3174 GP of control cells was significantly lowered by conventional CDs, but not the inverted derivatives (Figure 3C).

Furthermore, to characterize the time-dependence of changes induced by MβCD, HPβCD and HPγCD in membrane fluidity, we repeated our measurements with TMA-DPH by pre-labeling cells with the fluorophore, which was followed by incubating them with 5 mM CDs in the cuvette used in the spectrofluorometer. Treatments started immediately before the initiation of measurements that included repeated quantification of fluorescence anisotropy every minute in the first 10 min of incubation and, as an end-point of the experiments, after 60 min. Consistent with results of our kinetic cholesterol quantitation experiments, time-dependent reductions were observed in fluorescence anisotropy values for all three examined CDs (Figure 3D). Membrane fluidizing effects of MβCD and HPβCD were similar and much higher in magnitude than those of HPγCD. For MβCD and HPβCD, significant decreases occurred after 8 min, while in the case of HPγCD reductions were not significant in the first 10 min. Based on the kinetic traces it could be concluded that only relatively small, 15–20% reductions occurred in the first 5 min. These data suggest that alterations in membrane biophysical parameters can be expected in response to cholesterol-extracting CDs mainly after longer incubation periods not in the time scale of patch-clamp experiments.

### Potential Mechanisms of Cyclodextrin Actions on K_V_1.3

**FIGURE 4:**
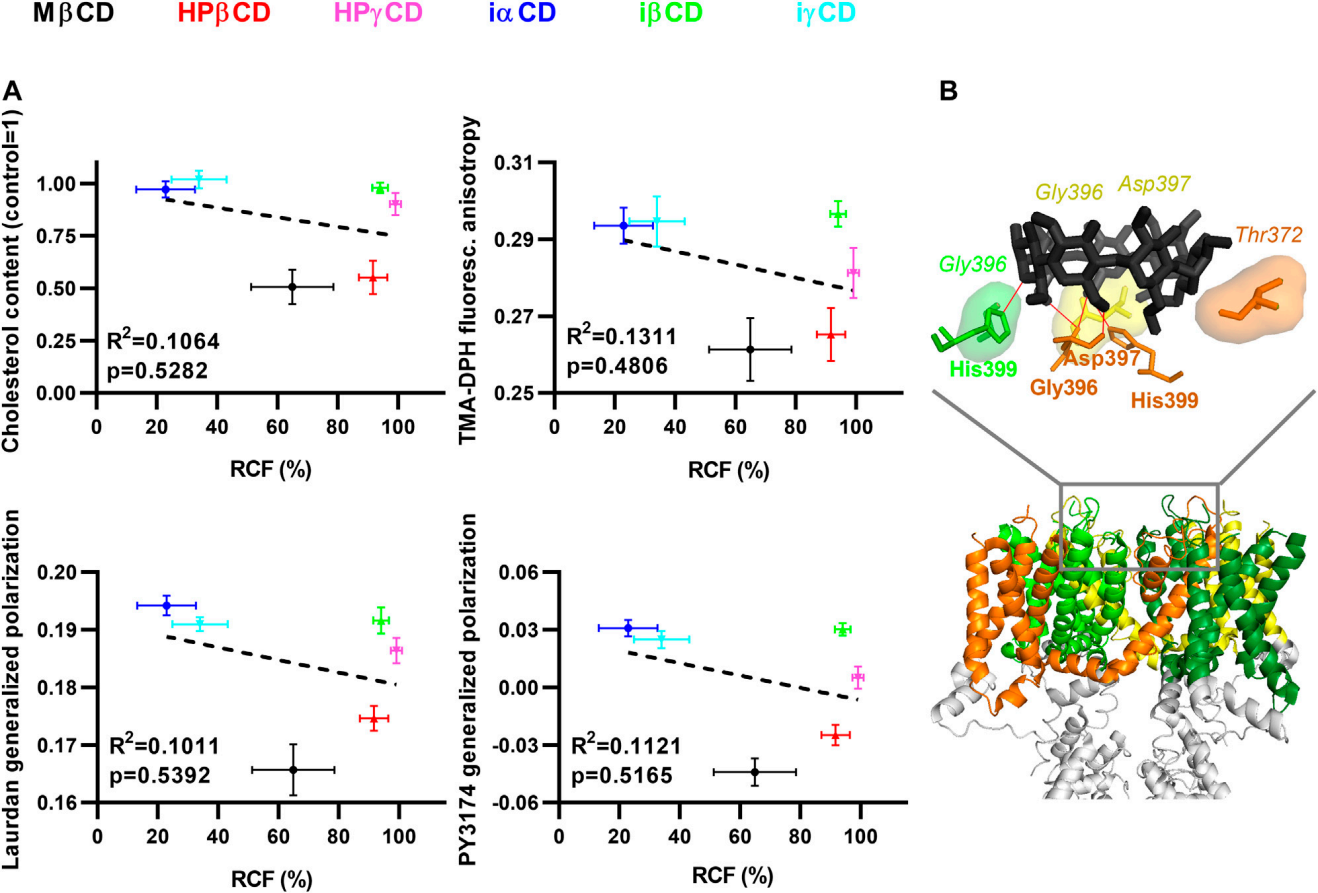
Potential mechanisms of CD actions on K_V_1.3. (**A**) To correlate changes between electrophysiological parameters, cholesterol levels and membrane biophysical parameters, CHO cells were treated with 5 mM MβCD, HPβCD, HPγCD, iαCD, iβCD or iγCD for 1 h, which was followed by determination of K_V_1.3 remaining current fraction (RCF), cholesterol level, TMA-DPH fluorescence anisotropy, and Laurdan and PY3174 generalized polarization (GP), as described previously in detail. Average values (±SEM) of cholesterol level, TMA-DPH fluorescence anisotropy, Laurdan GP and PY3174 GP were plotted as function of RCF values. *R* ^2^ and *p* values determined with linear regression analysis are shown in the panels, which revealed no significant correlation between RCF and any of the other examined parameters. (**B**) To examine possible direct interactions between CDs and K_V_1.3, *in silico* molecular docking was performed with AutoDock Vina using MβCD (from structure PDB 2QKH (black)) and the cryo-electron microscopic structure of Kv1.3 (PDB 7EJ1). S1-S6 transmembrane helices of different subunits are shown in different colors, while the rest of the channel (tetramerization domain) is displayed in grey. One representative binding mode of MβCD to the extracellular surface of the pore domain is shown in the upper panel. Amino acids taking part in ligand-target interactions were identified and displayed using LigPlot+ and PyMol. The identified hydrogen bonds are illustrated with red lines, while hydrophobic interactions are labeled by shaded areas between MβCD and K_V_1.3. Amino acid residues taking part in hydrogen bonds are shown in bold, while those involved in hydrophobic interactions are represented in italic.

To quantitatively characterize the correlation between K_V_1.3 current-inhibiting propensities of the examined CDs and their abilities to deplete membrane cholesterol and modify membrane biophysical parameters, we performed linear regression analysis on results described in previous sections, which were obtained after 1-h incubation in the presence of 5 mM CDs. *R* ^2^ and *p* values revealed no significant correlation between RCF values determined in patch-clamp measurements and cholesterol levels, TMA-DPH fluorescence anisotropy, Laurdan GP or PY3174 GP values (Figure 4A), further supporting the existence of a cholesterol-independent current-blocking effect of certain CDs.

Furthermore, to examine potential direct interactions between CDs and K_V_1.3, we performed *in silico* molecular docking analysis between MβCD and Kv1.3 (Figure 4B). Since electrophysiological evidence argues against binding of MβCD intracellularly (current-inhibiting effects of all CDs appeared in 15 s and completed within 90 s) or to the voltage-sensor (no shift in activation threshold, data not shown), the extracellular orifice of the pore domain was chosen as a search space for docking covering the most frequent binding sites of toxins and small molecule inhibitors (Kavanaugh et al., 1991; Holmgren et al., 1997; Gilquin et al., 2005; Karbat et al., 2019) (Supplementary Figure S1). Ten binding modes were recorded, which were characterized by estimated K_d_s consistent with the dose-dependence of current inhibition described above (binding affinities were in the range of −4.3 to −3.9 kcal/mol corresponding to estimated K_d_ values between 618 and 1,229 µM). These modes identified a common pattern of direct binding involving a network of hydrogen bonds and hydrophobic interactions between MβCD and pore residues. The association was typically mediated by hydrogen bonds formed by His399, Gly396 and Thr373 or Asp397, and hydrophobic interactions through Asp397, Gly375 or Tyr395 of one subunit. The structure was usually further stabilized by a hydrogen bond with His399 and hydrophobic interaction with Gly396 on the adjacent subunit, and additional hydrophobic interactions with Gly396 and Asp397 of the opposing subunit (Figure 4B).

## Discussion

Due to their unique chemical structure providing the basis for forming water-soluble complexes of otherwise non-soluble lipids and drugs, CDs are extensively used in both research and clinical applications (Davis and Brewster, 2004; Uekama, 2004; Loftsson et al., 2005; Szente and Fenyvesi, 2017; Szente et al., 2018). According to *in vitro* studies (Szente and Fenyvesi, 2017; Szente et al., 2018) and our experiments as well, MβCD has the highest affinity to deplete cholesterol from the membrane of living cells (Figure 1), which ensures its popularity in the laboratory practice. On the contrary, MβCD cannot be applied parenterally in clinical practice due to its *in vivo* hemolytic effect (Irie et al., 1982; Ohtani et al., 1989). Thus, HPβCD is the first choice to deplete membrane cholesterol in medical practice due to its increased biological tolerability (Irie and Uekama, 1997; Szente et al., 2018). HPβCD got recently into the focus of many ongoing clinical trials since it received an orphan drug status for the treatment of NPC disease by Food and Drug Administration (Matencio et al., 2020), further corroborating the increasing potential of CDs in therapeutic applications.

MβCD alone or pre-complexed with cholesterol is typically employed for 1 h with concentrations ranging from 3 up to 10 mM (Zidovetzki and Levitan, 2007). Local CD concentrations of comparable magnitude can also be reached in medical use. For example, due to its poor blood-brain barrier penetration ability and short biological half-life, the dosage of HPβCD in the treatment of NPC disease is extremely high (1,200–2,500 mg/kg/week) (Megias-Vericat et al., 2017; Carradori et al., 2020). This protocol leads to the incidence of severe side effects including chemical meningitis and sensorineural hearing loss (Crumling et al., 2017; Megias-Vericat et al., 2017; Hammond et al., 2019). While both beneficial and adverse effects of CDs are generally thought to originate from their cholesterol-extracting actions, direct interactions with K_V_ channels might also contribute to their effects.

CDs can influence the function of proteins in two distinct ways. On one hand, as the structure and function of transmembrane proteins are substantially influenced by cholesterol, CDs can modify protein functions through alterations in cholesterol levels and related membrane biophysical parameters (Zakany et al., 2020). On the other hand, CDs can affect proteins through direct, ligand-like interactions mediated through binding to their aromatic amino acid residues including tyrosine, phenylalanine, tryptophan and possibly histidine as demonstrated recently for many proteins (Aachmann et al., 2003; Prior et al., 2007; Wahlström et al., 2012; Ren et al., 2016; Dai et al., 2017). In these cases, the suggested mechanism involves mainly hydrophobic and van der Waals interactions resulting in the inclusion of these residues into the hydrophobic cavity of CDs, and the association properties are determined by shape-matching and optimum hydrophobicity conditions (Gautam et al., 2017; Niccoli et al., 2017). Furthermore, CDs were recently proposed to also interact with other amino acids including Asp, Asn, Lys or even His through hydrogen bonds, and the binding orientation of CD might depend on its possible chemical substitutions and the given interacting residue (Banerjee et al., 2010). Numerous studies investigated the effects of CDs on K_V_1.3, a prototypical and pathophysiologically relevant K_V_ channel, substantially affected by cholesterol (Hajdú et al., 2003; PottosinValencia-Cruz et al., 2007; Balajthy et al., 2016; Zakany et al., 2019; Zakany et al., 2020), however these studies focused solely on cholesterol-mediated actions and examination of direct interaction between CDs and K_V_1.3 has not been reported yet. In this aspect, interaction between CDs and K_V_s might be similar to those between polyunsaturated fatty acids and these channels. Although the effect of polyunsaturated fatty acids have long been considered to be mediated indirectly by modulating the biophysical properties of membranes, recent studies demonstrated direct binding of these lipids to members of the K_V_7 family leading to substantial alterations in its electrophysiological parameters (Liin et al., 2018; Yazdi et al., 2021).

In this study we report on direct, ligand-like inhibitory effect of CDs on the K_V_1.3 ion channel at biologically relevant millimolar concentrations, which is independent of membrane cholesterol depletion and concomitant alterations in membrane biophysical parameters caused by CDs. Based on our results, this hypothesis is supported by the following arguments: 1) According to literature data cholesterol depletion by 1-h MβCD treatment results in an increase in K_V_1.3 current, while we detected current inhibition by MβCD completed within 90 s (Figures 2C,E). 2) Cholesterol-depleting and current-inhibiting abilities of CDs are not in parallel since MβCD, iαCD and iγCD were able to block Kv1.3 currents (Figures 2C,D,E), while only MβCD exhibited significant cholesterol extraction after longer exposure (Figure 1B and Figure 4A). On the other hand, cholesterol-depleting HPβCD and HPγCD (Figure 1) did not inhibit currents (Figures 2C,E, Figure 4A). 3) Similarly to cholesterol extraction, K_V_1.3 current inhibition showed no correlation with alterations in membrane biophysical parameters including fluidity, hydration and lipid order (Figure 2E, Figure 3 and Figure 4A). 4) Ion channel blockade in response to cholesterol-extracting MβCD was completed on a time-scale where no significant changes were observed in membrane cholesterol level or fluidity (Figure 1B, Figure 2C and Figure 3D). 5) Current inhibitory effects of MβCD, iαCD and iγCD were dose-dependent at concentrations that are comparable in magnitude with those reached in blood during parenteral administration of CD-containing medications or when used in research to modify membrane cholesterol levels (Figure 2E). 6) Consistent with our electrophysiological data, molecular docking analysis revealed potential direct interactions between MβCD and the extracellular part of the pore domain of K_V_1.3 (Figure 4B).

Although the patch-clamp measurements did not shed light on the binding site of CDs in K_V_1.3, the kinetics of current block may provide a hint whether they bind to an intra- or extracellular location. According to our results the blocking effects of the tested CDs appeared in 15 s and completed within 90 s (Figures 2C,D). Given that CDs are generally considered to enter cells via endocytic mechanisms (Rosenbaum et al., 2010; Fenyvesi et al., 2014), this suggests that CDs are more likely to bind to the extracellular surface of the channel. The generally known pore blockers have three potential binding regions on K_V_ channels: the intracellular cavity (residue Ile420 in K_V_1.3) (Holmgren et al., 1997), the extracellular mouth of the pore (residue His399) (Kavanaugh et al., 1991; Gilquin et al., 2005), and the turret region (residue Gly375) (Karbat et al., 2019). Supporting direct binding of CDs to the extracellular surface of the pore domain of the channel, our molecular docking analysis identified potential MβCD binding sites organized mainly by His399 of neighboring subunits (Figure 4B), however, interactions of the identified residues with other CDs and their physiological relevance should be tested by future studies applying specific mutations. Furthermore, our results do not provide an unequivocal mechanism of CD effects, i.e., whether CDs directly plug the orifice of the pore or rather induce a conformational change resulting in the block of ion conduction. Thus, inhibitors selectively interacting with these sites could provide additional relevant information to fully characterize the mechanism of direct current-inhibiting actions of CDs on K_V_ channels. Interestingly, while iαCD and iγCD efficiently blocked K_V_1.3, iβCD failed to inhibit ion conduction. Although a reliable explanation for the distinctive behavior of iCDs is not possible based on our findings, it can originate from differences in their size and hydrophobicity profile, binding affinity, site and/or orientation, which can result in its inability to physically occlude the pore or induce conformational changes in this region leading to channel block.

Our results propose that direct blocking effects of CDs exerted on K_V_ channels may have physiological consequences during their clinical and research applications. In the future, identifying direct interactions of CDs with various proteins including other members of the K_V_ family and elucidating their mechanism of binding can provide significant improvements in the understanding of the beneficial and adverse effects of CD-containing medications and contribute to the chemical design of CDs with more favorable effect profile.

## Data Availability

The raw data supporting the conclusion of this article will be made available by the authors, without undue reservation.

## Abbreviations

AMP, adenosine monophosphate; ANOVA, analysis of variance; CD, cyclodextrin; CHO, Chinese hamster ovary cell; GP, generalized polarization, HPβCD, hydroxypropyl-β-cyclodextrin; HPγCD, hydroxypropyl-γ-cyclodextrin; iCD, ‘inverted’ Per(3,6-anhydro)-cyclodextrin; iαCD, hexakis (3,6 anhydro)-α-cyclodextrin; iβCD, heptakis (3,6 anhydro)-β-cyclodextrin; iγCD, octakis (3,6 anhydro)-γ-cyclodextrin; K_V_, voltage-gated potassium channel; Laurdan, 6-dodecanoyl-N,N-dimethyl-2-naphthylamine; MβCD, methyl-β-cyclodextrin; NBD, cholesterol: 25-[N-[(7-nitro-2-1,3-benzoxadiazol-4-yl)methyl]amino]-27-norcholesterol; NPC, Niemann-Pick type C disease; PY3174: di-4-AN(F)EPPTEA; 4-[2-(6-Dibutylamino-5-fluoro-naphthalen-2-yl)-vinyl]-1-(3-triethylammonio-propyl)-pyridinium dibromide; RCF, remaining current fraction; RF, recovered current fraction; TMA-DPH, 4′-(trimethylammonio)-diphenylhexatriene.
