# Supplementary material for: Cyclodextrins Exert a Ligand-like Current Inhibitory Effect on the KV1.3 Ion Channel Independent of Membrane Cholesterol Extraction
Source: Front Mol Biosci. 2021 Nov 4;8:735357. doi: 10.3389/fmolb.2021.735357 (PMC8599428; doi:10.3389/fmolb.2021.735357)
Supplement: Supplementary file 1 [file DataSheet1.PDF]

Supplementary Material

1 Supplementary Methods

Transfection of CHO cells

To induce expression of Kv1.3 channels, CHO cells cultivated according to their specifications were co-transfected with wild-type Kv1.3 and enhanced green fluorescent protein (EGFP) encoding plasmids (OriGene Technologies, MD, USA) at a mass ratio of 10:1 using Lipofectamine2000 (Thermo Fisher Scientific, Waltham, MA) at a lipid to DNA ratio of 2:1 (µl/µg) according to the manufacturer's protocol. Channels were transiently expressed in cells for 12–48 h.

Additional characterization of the applied CDs

Symbol	iαCD	iβCD	iγCD
Chemical name	Hexakis(3,6-anhydro)-α-cyclodextrin	Heptakis(3,6-anhydro)-β-cyclodextrin	Octakis(3,6-anhydro)-γ-cyclodextrin
Molecular mass	864	1008	1120
Optical rotation	$[\alpha]_D^{25}$-82.5 (air-dry substance)	$[\alpha]_D^{25}$-33 (0.1 m, water 2%)	$[\alpha]_D^{25}$-92.5 (0.1 m, water 2%)
Melting point	230-235 °C	241-246 °C	230-235 °C
Solubility	soluble in water	soluble in water	soluble in water
Conductivity of 1% aq. solution	25 µS/cm	28 µS/cm	28 µS/cm
Chemical purity by HPLC	98.1%	97.3%	97.3%
Chemical structure	confirmed by NMR spectroscopy		

Patch-clamp measurements

For data acquisition a Multiclamp 700B amplifier attached to a personal computer (Digidata 1440A data acquisition hardware, Molecular Devices, Sunnyvale, CA) was employed. Series resistance compensation up to 70% was used to achieve good voltage-clamp conditions and minimize voltage errors. EGFP-positive transfectants were identified on a Nikon Eclipse TS-100 fluorescence microscope (Nikon, Tokyo, Japan) using bandpass filters of 455–495 nm and 515–555 nm for excitation and emission, respectively. Pipettes were pulled from GC 150 F-15 borosilicate glass capillaries (Harvard Apparatus, Kent, United Kingdom) in five stages, which resulted in electrodes with 3–5 MOhm resistance in the bath.

Pipette solution contained (in mM) 105 KF, 35 KCl, 10 HEPES and 10 EGTA and was titrated to pH 7.36–7.38 with KOH, with a final K^+ concentration of 160–165 mM and osmolarity of 285–295 mOsm/L. Standard extracellular solution was composed of (in mM) 150 NaCl, 2 KCl, 1.5 $CaCl_2$, 1 $MgCl_2$, and 10 HEPES. The osmolarity of the solution was 290 mOsm/L, and pH was titrated to 7.36–7.38 with NaOH. The high potassium containing extracellular solution used for validating the perfusion system was composed of (in mM) 150 KCl, 10 HEPES, 5.5 glucose, 2.5 $CaCl_2$ and 1 $MgCl_2$.

Data were analyzed using Clampfit (v10; Molecular Devices) and SigmaPlot (v10.; Systat Software, San Jose, CA). Current traces were adjusted for ohmic leakage before analysis and were digitally filtered using a three-point boxcar smoothing.

Examination of membrane biophysical parameters

To measure membrane fluidity and hydration, cells treated with CDs for 1 hour were labeled with 10 μM 4′-(trimethylammonio)-diphenylhexatriene (TMA-DPH) or 2 μM 6-dodecanoyl-N,N-dimethyl-2-naphthylamine (Laurdan, both from Sigma-Aldrich) for 20 min at room temperature, then fluorescence intensities were measured with a Fluorolog-3 spectrofluorometer (Horiba Jobin Yvon, Edison, NJ) at 37 °C. Alternatively, for kinetic measurements of membrane fluidity, cells were pre-labeled with TMA-DPH and CD treatment started in the cuvette holder immediately before initiation of the measurement. Anisotropy was repeatedly evaluated every 60 s in the first 10 minutes of treatment.

The fluorescence anisotropy (*r*) of TMA-DPH was determined after an excitation at 352 nm and measurement of fluorescence at 430 nm:

$$r = \frac{I_{vv} - GI_{vh}}{I_{vv} + 2GI_{vh}} \quad (1),$$

where I_{vv} and I_{vh} are the vertical and horizontal components, respectively, of the fluorescence excited by vertically polarized light, and G is an instrument-specific correction factor characterizing the different sensitivity of the detection system for vertically and horizontally polarized light.

Laurdan was excited at 350 nm and its emission was detected in two ranges (at 435 nm – I_{blue} and 500 nm – I_{red}). Generalized polarization (GP) was calculated as:

$$GP = \frac{I_{blue} - I_{red}}{I_{blue} + I_{red}} \quad (2).$$

PY3174 (di-4-AN(F)EPPTEA; 4-[2-(6-Dibutylamino-5-fluoro-naphthalen-2-yl)-vinyl]-1-(3-triethylammonio-propyl)-pyridinium dibromide), a kind gift from Leslie M. Loew (University of Connecticut, CT) (1), was applied to quantify lipid order as previously (2). Cells were treated with CDs for 1 hour and stained with 10 μM PY3174 for 20 min at room temperature and images were taken at the midplane of cells using an LSM880 confocal laser-scanning microscope (Carl Zeiss AG, Jena, Germany). PY3174 was excited at 488 nm and emitted intensities were measured in two wavelength ranges between 500 and 540 nm (I_{blue}) and 630 and 735 nm (I_{red}). During processing, the average value of GP was determined from the data of cell membrane pixels identified with a manually seeded watershed algorithm with a custom-written Matlab program (3) after background subtraction using equation (2).

2 Supplementary Figure

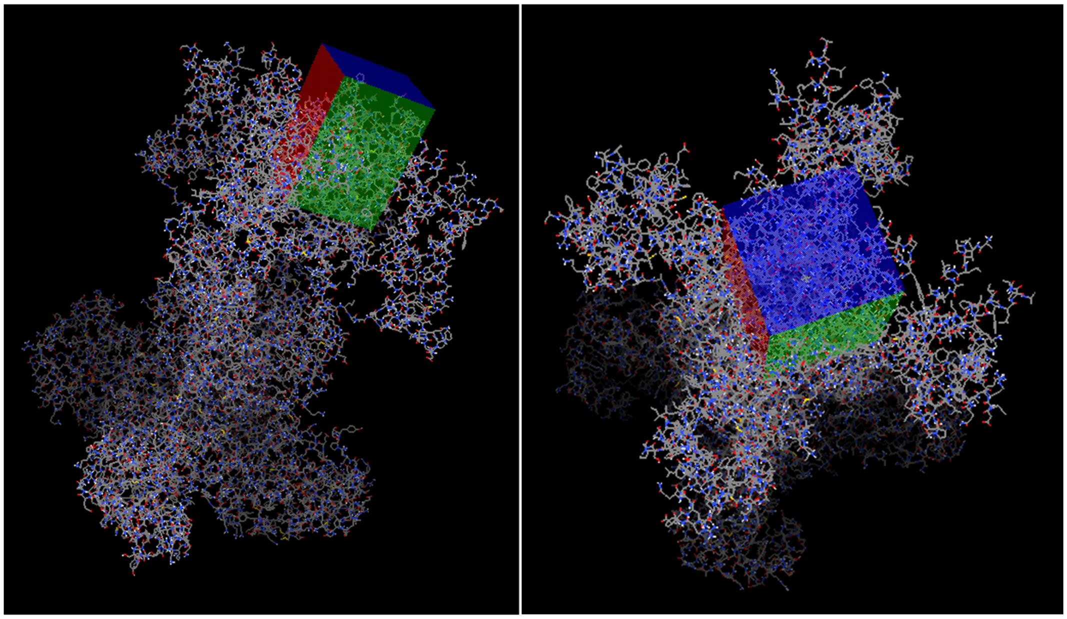

Supplementary Figure 1. Selection of the search space for molecular docking analysis between MβCD (from PDB 2QKH) and Kv1.3 (PDB 7EJ1). Given that pore region of Kv1.3 contains the most frequent binding sites for toxins and small molecule inhibitors blocking the channel, and the results of our electrophysiological experiments argued against binding of MβCD intracellularly or to the voltage-sensor domain of the channel, the search space was defined to include the extracellular orifice of the pore, which is displayed by the colored cuboid overlaid on the structure of Kv1.3.

3 Supplementary References

1. Kwiatek JM, Owen DM, Abu-Siniyeh A, Yan P, Loew LM, Gaus K. Characterization of a new series of fluorescent probes for imaging membrane order. *PLoS One* (2013) 8(2):e52960. doi: 10.1371/journal.pone.0052960. PubMed PMID: 23390489; PubMed Central PMCID: PMCPMC3563652.

2. Zakany F, Szabo M, Batta G, Karpati L, Mandity IM, Fulop P, et al. An omega-3, but Not an omega-6 Polyunsaturated Fatty Acid Decreases Membrane Dipole Potential and Stimulates Endo-Lysosomal Escape of Penetratin. *Front Cell Dev Biol* (2021) 9:647300. doi: 10.3389/fcell.2021.647300. PubMed PMID: 33912562; PubMed Central PMCID: PMCPMC8074792.

3. Kovacs T, Batta G, Zakany F, Szollosi J, Nagy P. The dipole potential correlates with lipid raft markers in the plasma membrane of living cells. *J Lipid Res* (2017) 58(8):1681-91. doi: 10.1194/jlr.M077339. PubMed PMID: 28607008; PubMed Central PMCID: PMCPMC5538289.
